# 

**Published:** 1849-07

**Authors:** 


### BOOKS RECEIVED.

Through Messrs. Beckwith & Morton, booksellers of this city, we have received from the publishers, Messrs. Lindsay & Blakiston, an interesting volume on Anesthesia, from the pen of Prof. Simpson, of Edinburg; and the neat and compendious work of Noad on Chemical Analysis, qualitative and quantitative. From Messrs. Barrington & Haswell we have received, through the same channel, the late edition of Gooch's Compendium of Midwifery; and to Messrs. Lea & Blanchard we are indebted for a copy of Mohr, Redwood and Procter's Pharmacy. Fuller notices of these works will appear as early as we can find room for them.

### RECEIPTS OF MEDICAL JOURNAL FOR JUNE 1849.

| | |
|---|---|
| Dr. Yager, county, | $2 50 |
| J. A. Brown, Warsaw, Mo., | 10 00 |
| J. C. Wilson, Flemingsburg, Ky., | 5 00 |
| W. S. C. White, Glasgow, Ky. | 5 00 |
| J. H. Sloan, Jackson, Ark. | 5 00 |
| W J. Routh, Carrolton, Ark. | 5 00 |
| Robinson, county, | 5 00 |
| —— Rucker, Huntsville. Mo., | 5 00 |
| —— Dameron, Huntsville, Mo., | 5 00 |

